# Pseudotumour Cerebri Induced by All-Trans Retinoic Acid: A Case Report

**DOI:** 10.7759/cureus.48520

**Published:** 2023-11-08

**Authors:** Vivek Ramburuth, Leila Khalil

**Affiliations:** 1 Endocrinology & Diabetes, Peterborough City Hospital, Peterborough, GBR; 2 Haematology, Peterborough City Hospital, Peterborough, GBR

**Keywords:** acute promyelocytic leukaemia, tretinoin, idiopathic intracranial hypertension (iih), pseudotumour cerebri, arsenic trioxide (ato), all-trans-retinoic acid

## Abstract

Acute promyelocytic leukaemia (APL) is an aggressive type of leukaemia associated with severe coagulopathy and haemorrhage. Treatment with all-trans retinoic acid (ATRA) combined with arsenic trioxide (ATO) therapy is life-saving and induces excellent remission rates. However, ATRA can, on rare occasions, cause pseudotumour cerebri, which is characterised by an elevation in intracranial pressure without evidence of infection or vascular or structural abnormalities. We describe a case of pseudotumour cerebri that was precipitated by ATRA therapy. Intracranial hypertension should always be considered in patients with headaches and visual complaints with normal neuroimaging. Early identification and management are essential to preventing visual loss.

## Introduction

Acute promyelocytic leukaemia (APL) is an aggressive subtype of acute myeloid leukaemia. It is defined by the presence of the promyelocytic leukemia-retinoic acid receptor (PML-RAR) alpha fusion gene produced by a translocation between chromosomes 15 and 17 [1]. The features of APL include a younger age of onset, the presence of leucopenia, and a high incidence of severe coagulopathy and haemorrhage [2,3]. The standard of care for low- and intermediate-risk APL is all-trans-retinoic acid (ATRA) and arsenic trioxide (ATO) dual differentiation therapy [4]. A randomised controlled trial demonstrated that ATRA-ATO combination therapy can achieve remission rates of 100% [5]. However, these agents are associated with side effects, and on rare occasions, ATRA can cause pseudotumour cerebri, also known as idiopathic intracranial hypertension [6].

Pseudotumour cerebri is characterised by an elevation in intracranial pressure without evidence of infection or vascular or structural abnormalities [7]. It is a rare disorder that affects one to three people in every 100,000 [8]. It may be a primary disorder, predominantly seen in obese female patients of childbearing age, or it may be secondary to various medical conditions (Addison's disease, hypoparathyroidism, obstructive sleep apnea), obstruction of the venous system (cerebral venous sinus thrombosis, jugular vein thrombosis), medications (steroids, lithium, amiodarone), or infections (HIV, borreliosis) [9].

We describe a case of pseudotumour cerebri that developed shortly after commencing induction therapy for APL with ATRA and ATO. This article was previously presented as a poster at the Royal College of Physicians Update in Medicine-Cambridge conference on May 11, 2023.

## Case presentation

Medical history and demographics

A 30-year-old female presented to her general practitioner with fatigue and lethargy. She had an elevated body mass index (BMI) of 40.1 kg/m^2^ and no other medical history. She was not on chronic medications, was a non-smoker, and did not drink alcohol. A full blood count showed a low neutrophil count, and a peripheral blood film revealed the presence of promyelocytes. Fluorescence in situ hybridization analysis of a peripheral blood sample was positive for the PML-RAR alpha fusion gene, confirming a diagnosis of APL.

She was started on induction therapy with ATRA at 45 mg/m2 (in two divided doses) and ATO. After 12 days of therapy, she developed a persistent throbbing headache associated with photophobia and double vision. On neurological examination, significantly reduced visual acuity and bilateral cranial nerve VI palsy were noted. Fundoscopy revealed bilateral papilloedema with blurred optic disc margins. Visual field testing using the confrontation method was normal; pupils were equally reactive, and the remaining cranial nerves were normal. There was no nuchal rigidity, and Kernig’s sign was negative. Upper and lower limb examinations were unremarkable.

Investigations

Her laboratory data are shown in Table 1.

**Table 1:** Summary of the patient's laboratory data CSF: cerebrospinal fluid

	Value	Reference range
Blood test		
Haemoglobin (g/L)	84	130-180
White cell count (10^9^/L)	1.6	4.0-11.0
Platelet count (10^9^/L)	125	150-400
Activated partial thromboplastin time (seconds)	26	24-36
Prothrombin time (seconds)	13	9-16
Fibrinogen (g/L)	3.4	2.0-4.5
C-reactive protein (mg/L)	2.0	<5
Sodium (mmol/L)	138	133-146
Potassium (mmol/L)	4.5	3.5-5.3
Creatinine (μmol/L)	65	59-104
Bilirubin (umol/L)	7	<21
Albumin (g/L)	32	35-50
Alkaline phosphatase (U/L)	68	30-130
CSF analysis		
Opening pressure (cm H₂O )	42	6-25
CSF white cell count per ul	<2	<5
CSF red blood cell per ul	18	0
CSF protein (g/L)	0.29	0.15-0.45
CSF glucose (mmol/L)	3.2	2.2-4.0
CSF culture	No growth	No growth
CSF cytology	No malignant cells	Acellular

Blood investigations showed the presence of anaemia and leucopenia, which were unchanged compared to the levels on admission. Her platelet count, coagulopathy tests, renal function, and liver function tests were unremarkable. A lumbar puncture was performed, and cerebrospinal fluid (CSF) analysis was unremarkable with normal protein, glucose levels, and cytological findings (Table 1). The CSF culture was negative. The CSF opening pressure was, however, markedly raised at 42 cm H₂O (normal 6-25 cm H₂O).

**Figure 1:**
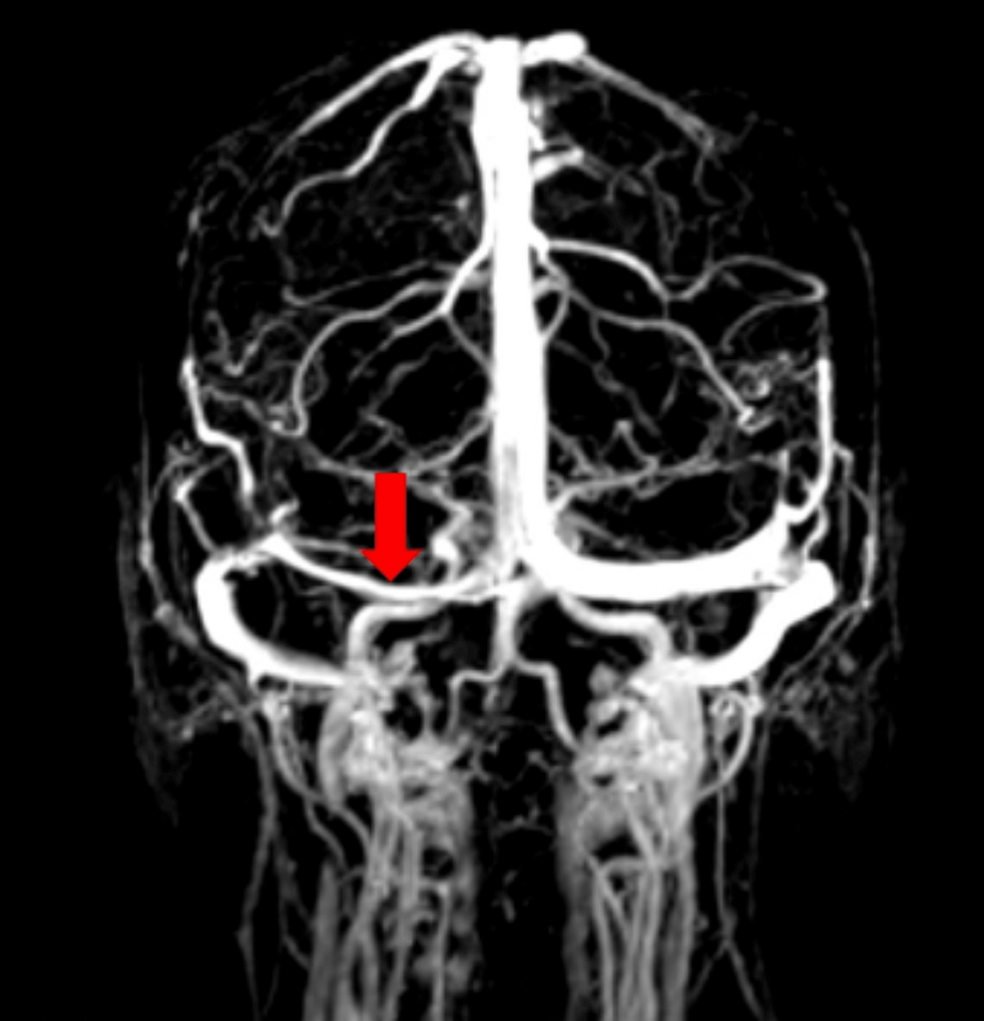
Magnetic resonance venogram showing smooth-walled narrowing of the transverse sinus (arrowhead)

A contrast-enhanced computed tomography scan of the head was performed and showed no evidence of intracranial metastases or leptomeningeal enhancement. A contrast-enhanced magnetic resonance scan of the brain was also unremarkable. A magnetic resonance venogram, however, revealed smooth-walled stenosis of the right transverse venous sinus (Figure 1).

**Figure 2:**
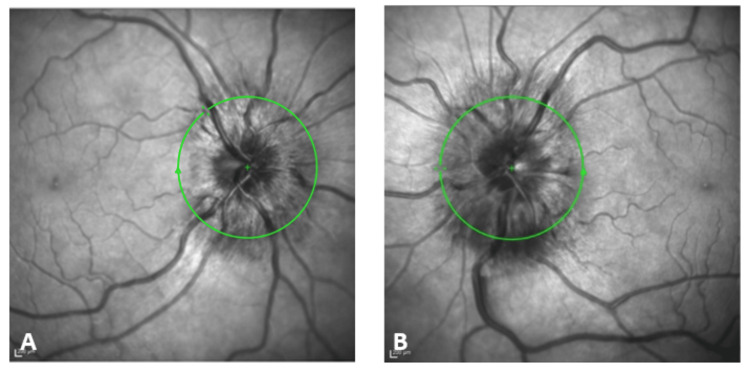
Optical coherence tomography of the right eye (A) and left eye (B) shows severe papilloedema and disc haemorrhage.

She was referred to the ophthalmologist, and optical coherence tomography confirmed the presence of severe bilateral papilloedema with engorged optic discs, blurred margins, and disc haemorrhage (Figure 2).

Management

The ATRA and ATO therapies were discontinued. Two therapeutic lumbar punctures were performed, and 25 ml of CSF was removed on both occasions, whereby a closing pressure of 17 cm H_2_O was achieved. She was also started on acetazolamide (250 mg) twice daily. After five days, her symptoms resolved, and a repeat ophthalmological assessment showed a reduction in her papilloedema. She was then rechallenged with ATRA at a lower dose (25 mg/m2), and ATO was resumed. She remained asymptomatic and was discharged.

Outcome and follow-up

She has since completed induction and four cycles of consolidation therapy alongside prophylactic acetazolamide (250 mg, twice daily). She did not develop further neurological or ocular symptoms and has remained in remission from her APL.

## Discussion

This patient developed pseudotumour cerebri shortly after commencing ATRA and ATO combination therapy. Several factors could potentially predispose her to developing intracranial hypertension, including her history of obesity, female gender, and age. All-trans-retinoic acid is well described as being associated with intracranial hypertension. In a study, it was noted that among 240 patients receiving ATRA therapy, 1.7% of cases developed pseudotumour cerebri [10]. A much higher incidence was noted in a case series by Smith MB et al., where 50% of the 10 patients receiving ATRA-ATO therapy developed the condition [11]. The authors suggested that ATO may foster the development of pseudotumour cerebri, leading to the high incidence rate observed [11]. The pathophysiology of ATRA-induced intracranial hypertension is unclear. All-trans-retinoic acid, a derivative of vitamin A, is thought to elevate intracranial pressure by altering the lipid constituents of the choroid plexus and arachnoid villi, leading to enhanced CSF production and impaired absorption [12]. Other forms of vitamin A, such as isotretinoin (Accutane), have similarly been associated with intracranial hypertension [12].

The diagnostic criteria for pseudotumour cerebri include the presence of papilloedema, an elevated CSF pressure >25 cmH2O, normal neurological examination, normal CSF constituents, and normal neuroimaging [7]. The diagnostic process requires the exclusion of important causes of intracranial hypertension in APL, such as venous sinus thrombosis, intracranial haematoma, bacterial meningitis, and leukemic invasion of the meninges. The neurological examination is typically normal, except for findings of papilloedema and sixth nerve palsy [13]. Although normal imaging is a prerequisite for establishing a diagnosis of pseudotumor cerebri, some imaging features are associated with intracranial hypertension, including flattening of the globe, an empty sella, and enlargement of the optic nerve sheath complex [14]. In this case, a magnetic resonance venogram revealed a narrowing of the transverse venous sinus. A similar finding was noted bilaterally in 94% of patients with idiopathic intracranial hypertension in a study by Morris et al. [15]. The authors suggested that transverse sinus stenosis is the most useful and sensitive imaging indicator of intracranial hypertension [15]. Lumbar puncture with opening pressure measurement is critical in making a diagnosis of pseudotumour cerebri. This can, however, be challenging in APL given the high rates of associated thrombocytopenia and coagulopathy.

Management strategies include discontinuation of ATRA and ATO, as well as other medications such as triazole antifungals, which influence the cytochrome P-450 system and may exacerbate intracranial hypertension [11, 16, 17]. In about a third of these patients, pseudotumour cerebri will resolve when ATRA is held with no further treatment [16]. High-volume therapeutic lumbar punctures, inhibition of CSF production with acetazolamide, and weight loss are widely used and successful strategies for managing intracranial hypertension [18]. In the context of ATRA-induced pseudotumour cerebri, withdrawal of ATRA therapy and reintroduction at a lower dose alongside prophylactic administration of acetazolamide is a useful treatment approach [16]. Early management is key to the prevention of visual loss from optic atrophy [19].

## Conclusions

Pseudotumour cerebri is an important complication that should be recognised in APL patients undergoing therapy with ATRA and ATO. In the context of neurological symptoms with normal neuroimaging, pseudotumour cerebri should be included in the differential diagnosis. Withdrawal of ATRA therapy and reintroduction at a lower dose alongside acetazolamide is a useful therapeutic approach. Early recognition and management are key to the prevention of visual loss.
